# Non-Contact Diagnostics of the Geometry of a Historic Wooden Building as an Element of Periodic Safety Assessment

**DOI:** 10.3390/s22041301

**Published:** 2022-02-09

**Authors:** Tomasz Lipecki

**Affiliations:** Faculty of Mining Surveying and Environmental Engineering, AGH University, Al. A. Mickiewicza 30, 30-059 Krakow, Poland; lipecki@agh.edu.pl

**Keywords:** deformation analysis, laser scanning, 3D reconstruction, geometric condition, structure safety, non-contact diagnostics

## Abstract

The article presents a method of non-invasive diagnostics of a historic wooden church, built in the 18th century. Over the hundreds of years of its use, changes in the geometry of the structure have been observed. This article presents the requirements of so-called architectural and geodetic survey and the method of using terrestrial laser scanning to create a three-dimensional solid model of an object. The diagnostic tests performed made it possible to perform analysis based on a so-called point cloud, which is a virtual representation of a real object. In order to determine the basic parameters of the building, the area and volume of all rooms were determined. It was found that the object exhibited deformations that cannot be explained solely as a result of imperfections during climb and normal wear and tear during operation. Therefore, the changes in shape were assessed in detail by means of an assessment of the verticality of the pillars supporting two levels of the church, the verticality of the walls, and the inconsistency of the floors, as well as the shape and horizontality of the roof edge. Additional InSAR and FEM tests of the object’s location on the ground allowed identification of the cause of the object deformation as the influence of inhomogeneous groundwater relations under the building. Without prophylactic measures, this deformation phenomenon can be expected to worsen. The tests described should therefore be considered as essential in subsequent diagnostic cycles and permit future extended numerical FEM analysis.

## 1. Introduction

The historical heritage of any nation is associated with buildings that testify to the cultural development and traditions of that country. One example which most clearly confirms this thesis is religious architecture. Poland possesses not only small Romanesque churches but also monumental Gothic, sophisticated Baroque, and wooden churches. These are a living testimony which has survived for hundreds of years despite the devastation of the country in many wars. For this reason, it is necessary to take great care of such buildings and ensure that they are preserved for future generations. One means of assessing structural stability is the analysis of the geometry of an object and its changes in terms of the study of structural deformation. This applies to both brick and wooden structures, the latter of which is a particular type of historical architecture preserved in Poland.

One of the methods for determining the structural deformations of the measured objects, also used in this research, is the finite element method (FEM). The geometric deviations of the structure identified during this test correspond to deteriorations and imperfections of the structure and allow to determine the predicted loads. FEM allows for a non-linear geometric analysis of the second or higher-order distribution of forces and stresses occurring in a deformed structure, which is possible according to the basic principles of the theory of elasticity and plasticity (e.g., [1,2]).

FEM is directly related to the objectives of this article, which presents a comprehensive, non-contact method of obtaining information for determining the boundary conditions of changes in structural geometry. The object described in the article is a wooden sacral building. Wooden structures are difficult to conduct analyses on using FEM, as well as geodetic measurements aimed at determining their deformation. In this case, it is particularly difficult due to the nature of the object (the monument); it is forbidden to perform contact tests, which limits the range of research methods used. This limitation presents the greatest challenge during diagnostic tests. The construction material also presents a challenge in terms of deformation assessment because wood is an anisotropic material and its properties vary depending on the direction of the force in relation to the arrangements of the core and fibres [3,4]. An important role in the assessment of its mechanical properties is played by reduction factors such as warping, buckling, element length scale, and creep, where the type of wood and its moisture content play a decisive role in the process of long-term deformation. Wooden structures are, in comparison with steel and reinforced concrete, particularly susceptible to ambient humidity and duration of load. For this reason, the advantages of a wooden structure (lightness, resistance to deformation, simplicity of erecting the structure, etc.) may be lost over the long-term, in particular if the wood has not been protected against harmful weather conditions or infestation. Thus, several-hundred-year-old wooden structures, despite the use of solid wood from the best types of trees, are subject to degradation resulting from imperfect protective techniques, and current restoration techniques and assessing changes in the geometry are not always able to restore constructions to their initial condition.

As mentioned above, conducting research in a historic church is very difficult. However, there are some technologies which can provide a non-contact way of obtaining geometry information, such as terrestrial laser scanning (TLS), which is popular nowadays [5]. TLS makes it possible to obtain a so-called point cloud representing the object from both the outside and the inside, along with its contents, and thereby meet the requirements of architectural surveys, which is one of the stages of building assessment. TLS is also often used for architectural surveys of sacral buildings. The aim of this kind of measurement is not only to faithfully reproduce the spatial layout but the interior design and technical and functional structure of architectural objects. It also aims to present the appearance of the object in a graphic but also descriptive form. This type of survey should contain materials and information that present the current state of the object. They constitute a historical document that can be used for works related to the protection of the facility or its reevaluation [6].

These types of surveys complement each other in providing reliable information about the building, which has been classified by Falkowski, Uchański, and Sörensen [7] into stages describing the purpose of the study, measuring, modeling the object, describing materials, and interpreting the results in the form of a semantic model.

The created measurement documentation enables the assessment of the technical condition of the object as well as the planning of maintenance and modifications. It also affords the opportunity to reconstruct elements of the object in case of damage or destruction. Conservators of monuments value the most accurate studies containing architectural details, as they allow planning of conservation. The introduction of the measuring technology presented in article [8]—laser scanning—allows texturing of a point cloud based on digital images or the intensity of the reflection of the laser beam, electronic levelling, and orientation of the instrument in the field (giving it global coordinates). Elements of the measured object such as edges and corners are not measured directly but must be modelled based on the measured point cloud. The result of a measurement carried out with the help of laser scanning may be the point cloud itself if elements of the scanner’s external orientation are determined in advance. From the point cloud, one can read distances, determine the coordinates of characteristic points, and generate cross-sections. Some existing standards allow only one form of presentation of analyses, namely the creation of a technical drawing. This can be made in the form of a three-dimensional vector model, provided that it contains all the information that is to appear in the two-dimensional drawing. The extension to 3D representation helps to create unified requirements for the description and presentation of an architectural survey carried out using terrestrial laser scanning.

For many years there has been discussion in the world literature on the possibility of using TLS in architectural, archaeological, and historical surveys. Examples of the possibilities of using TLS measurements can be found in articles describing the experiences of researchers in the measurements of ancient buildings [9,10,11]. These indicate that in order to obtain a correct result, an appropriately planned measurement procedure should be performed [12], but it is also necessary to constantly broaden knowledge of the possibilities of applying new measurement techniques [13]. As a result, it is possible, for example, to reconstruct the gothic vault of a chapel [14] or to recreate medieval castles [15,16] in digital form, even with the use of open-source software [17]. This enables ancient and modern times [18] to be connected virtually. To correctly recreate reality, the authors of [19] emphasize that geometry determined by measurements must be accurate and should provide a level of precision necessary for quantitative diagnostics. For this purpose, research on technology with the use of TLS has repeatedly been presented in terms of the accuracy of a point cloud, which reproduces measured objects in a virtual space. The authors of the publications [20,21,22,23,24] demonstrate that this technology in various forms is an excellent tool for the precise reconstruction of architectural objects and monuments, starting from small elements and ending with building complexes. Although we should take note of critical voices [25], which draw attention to the importance of supplementing such measurements with classic geodetic measurements (tacheometry, GPS) to obtain so-called georeferencing with an accuracy of more than 10 mm, this does not change the positive reception and increasingly widespread implementation of terrestrial laser scanning technology.

Such criticisms do indicate, however, the usefulness of multisensory approaches, i.e., research with the use of various types of measuring instruments, which show a synergy effect that allows a significant increase in knowledge about the object of study. The authors of [26] emphasize the importance of an integrated approach to the analysis and understanding of an archaeological context in order to document what is still tangible and to recreate both its original features and changes. One can then talk about 3D, 4D, etc., analysis, where the basis for locating information obtained from other measurement sensors are spatial data obtained by a geodetic technique (e.g., TLS). An example of such applications is the combination of TLS and mobile aerial scanning (ALS) [27,28], integration with the so-called texture mapping (photogrammetric techniques) [29,30], or the simultaneous use of portable X-ray fluorescence spectroscopy (pXRF), and photogrammetric techniques to evaluate monuments from the Byzantine era [31]. Thanks to such data acquisition, it is possible to create metric 3D models in the form of raw point clouds, but the biggest advantage is the possibility of reconstruction by 3D modelling using surface structures (triangulation and grid) and combinations of solids. Examples of this approach include the virtual reality modelling and visualization of Palermo Cathedral [32], the automatic algorithm for creating models of buildings [33] and objects such as historic monuments [34], and the three-dimensional visualization of the Palazzo Raimondi in Cremona [35]. Such modelling is not an end itself but can be used for construction purposes, enabling the reconstruction of the geometry of buildings as part of building information modelling (BIM), which is now becoming a comprehensive database of information about existing buildings (including historic ones) as well as designed objects. This is discussed in more detail in various publications such as [22,23,36].

Raw and processed laser scanning measurement data can be also used to determine the deformation of historic objects resulting not only from aging processes or devastation (in war) but also from the influence of the environment (earthquakes, wind pressure, floods, etc.). Examples of such analyses can be found in publications [24,35,37,38], in which the behaviour of a structure in a landslide region was examined, deviations from the ideal geometric condition were determined, or the load on arches and beam vaults was determined. Article [39] presents an analysis of a neo-Gothic church subjected to deformations in a mining area and discusses the problem of revealing deformations of brick structure. Concerning all the above information, these publications indicate the necessity and viability of using laser scanning to conduct advanced research based on spatial modelling. This is a case study on geometric diagnostics and deformation analysis and their causes for a wooden object as an element of structural research and preparation of input data for numerical analysis of structural statics. The approach is presented here in the form of comprehensive implementation of several types of analyses based on non-contact tests to identify the causes of object deformation.

## 2. Materials and Methods

### 2.1. Description of the Examined Church

**Figure 1:**
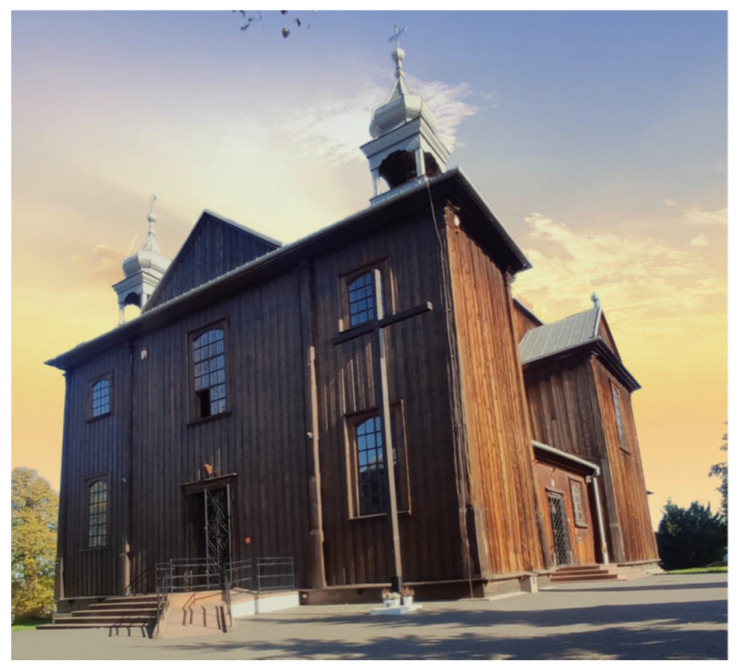
View of the church of St. Szczepan in Mnichów (source: private archive).

The subject of this article is a historic wooden church dedicated to St. Szczepan (Stephen) (Figure 1), located in Mnichów, a village in the Świętokrzyskie province of Poland. The building is located in a narrow area of Jurassic sea rock (lithologically: calcareous gaizes), which is covered with post-glacial sands, gravel, and clay. The western side of the church is crossed by the border of a third-order natural watershed, which also separates the geological mesoregions of the Nida River trough and the Jędrzejowska plateau. Such a location may be expected to have a negative impact on the stability of the foundation of the building. This architectural object is one of very few original wooden monuments that have been preserved to this day, as wood is a material susceptible to harmful biological and atmospheric factors. It has a dome with two towers in the facade and unique Rococo interiors, which have been maintained in a uniform style.

The date of the creation of this religious building is unfortunately unknown. Most sources state that it was erected in the 18th century. According to a study by Roman Mirowski of wooden churches and bell towers of the Świętokrzyskie region [40], the Mnichów church ceased to be a filial church of the Mokrsko parish in 1823, indicating that the church was already in existence at that time. Jaskłowski [41] claims that the church was built in 1731. On the other hand, Wiśniewski states in his work [42] that the church was built in 1754.

**Figure 2:**
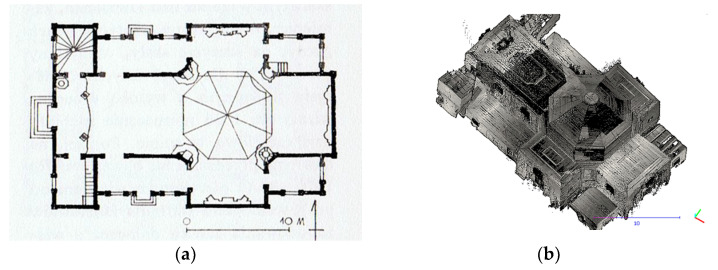
(**a**) Plan of the church of St. Szczepan in Mnichów (source: http://www.zabytkowekoscioly.net, accesed on: 15 October 2021) and (**b**) the scanned shape of the internal structure with a cross plan.

The construction of the church is very well thought out and architecturally homogenous. The first entries describing the church can be found in the Inventory fundi instructi [43]. They show that it was built of larch wood, covered with shingles, and had two entrances: a main entrance with pine doors, preceded by a stone staircase, and a smaller side entrance with a double door also made of pine. The floor in the church was made of boards. In the basement of the church there is a brick chapel, the vault of which is supported by columns and pillars. In 1903–1917, the roof covering was replaced with shingles of sheet metal. In 2014, conservation of damaged larch wood formwork began, but this did not change the existing geometric state of the building. The church has a log construction, which is covered with larch planks on the outside and is oriented with the altar wall facing east. It is laid out on a Latin cross plan (Figure 2a,b). It has a sacristy, two porches, and chapels on both sides of the presbytery. Above the intersection there is an octagonal dome, which has four rectangular windows in Baroque frames. Under the dome there is a slender octagonal lantern, which is covered with an onion-shaped helmet. The rectangular west facade is crowned with a triangular pediment surrounded by two four-sided towers with zinc sheet roofs with lanterns.

**Figure 3:**
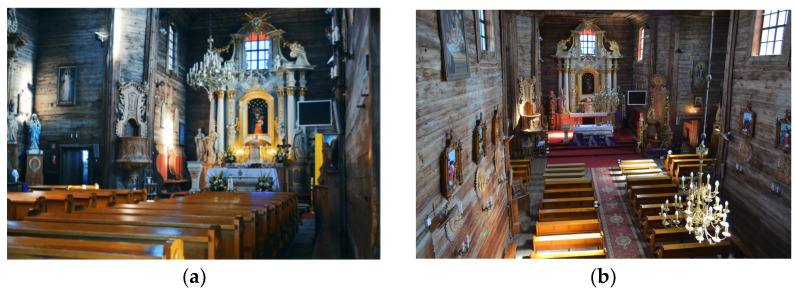
Interior of the church of St. Szczepan in Mnichów: view from the altar (**a**) and view from the choir on the altar (**b**); (source: private archive).

In the northern part of the transept a Rococo altar, entirely made of a larch wood, was constructed (Figure 3).

**Figure 4:**
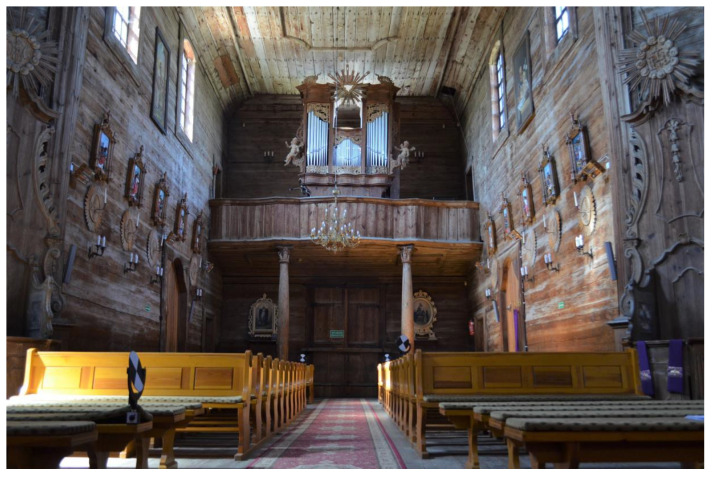
Music choir in the church of St. Szczepan in Mnichów (source: private archive).

On the western side of the interior there is a music choir (Figure 4). This is supported by slender, tall, wooden carved columns made in the Corinthian style. The organ prospectus placed on the choir is decorated with Rococo ornamentation.

The Mnichów church is a very valuable monument of Polish religious architecture. It is one of the few well-preserved wooden places of worship in Poland and is not only a historical monument but is still in use. However, the building requires systematic monitoring in which geodesic-architectural measurements play an important role. Unfortunately, such examination cannot involve physical contact with or disassembly of the object, which makes diagnostics and subsequent analysis difficult. For this reason, it was decided to use non-contact, ground-based laser scanning techniques, satellite interferometric synthetic radar aperture (InSAR), and finite element method (FEM), which in turn required the adaptation of existing diagnostic procedures to the specifics of the site, as described below.

### 2.2. Methods

Due to the limited conservation conditions and the possibility of conducting diagnostic tests in this type of historic buildings, the author proposed the use of the methodology that is based on the analysis of the largest possible set of information that can be obtained in limited conditions and multiple comparisons with the model that best describes it. For example, based on the experience of deformation analysis of objects in mining areas, it can be concluded that the image of this deformation is consistent with their causes (e.g., appropriate cracks in window and door openings, support deflection, floor and ceiling inclination, etc.). Thus, the distribution of deformation indicates potential (limited in the set) causes, which makes it easier to identify the specific cause of the deformation. For this reason, the disclosure of the appropriate set of deformations in the structure may narrow the analysis of the reasons for which those related to the instability of the subsoil are very important. Therefore, it was concluded that the hypothesis about the cause of the demonstrated deformations of the church are true if the premises, confirmed by various analyses (InSAR and FEM), indicate their cause in the same way. More about these analyses can be found in the publications [2,44]. In the event of inconsistency in inference from various methods, it is of course necessary to admit further working hypotheses, e.g., the effect of paraseismic shocks or construction faults.

Therefore, the proposed methodology is based on:

1. Diagnostics by a non-contact method (laser scanning);
2. Building a 3D model;
3. Selecting structural elements for which the current geometry is determined, and checking whether there are any deviations from the theoretical state;
4. If these deviations cannot be found, there are no premises confirming the hypothesis about object deformation under the influence of certain factors—the reasons are reclassified;

The hypothesis is confirmed on the basis of successive determinations of changes in geometry—in case of unequivocal inference about the cause of deformation, inference is made; otherwise, subsequent indications are selected in the form of the next typing of structural elements for analysis—return to point 3;

Completion of the analysis and confirmation with other methods and submission of final conclusions.

The methodology defined in this way, despite the limitation of the number of studies performed, makes it possible to substantiate the hypothesis about the cause of the object deformation under incomplete conditions of diagnostic tests.

#### 2.2.1. Geodetic Measurements

For the above methodology, it was decided to use a measuring device in the form of a laser scanner, which allows 3D assessment methods and is non-invasive in nature, which was also described in a number of publications [45,46]. The measurements of the wooden church of St. Szczepan in Mnichów were carried out using a Zoller & Froehlich Imager 5010C phase laser scanner. This made it possible to perform the tests to a very high accuracy (±5 mm), yielding a very detailed 3D representation of the measured object. This method of phase laser scanning means that a large amount of data can be obtained in a relatively short time, and it works in the near-infrared field. In the case of a historic building with a lot of old polychromes, this is a key consideration. Measuring a structure using a laser scanner involves measuring the distance d and the angle between the device and the target. Phase scanners use the phenomenon of a phase shift. They constantly send infrared waves towards the rotating mirror of the scanner. Infrared rays are reflected from the mirror and are directed towards the object being measured, from which they are reflected and return to the scanner. Based on the phase shift of the infrared radiation wave which returns to the scanner, the distance is calculated. For obtaining a set of distances d in 3D space, the laser signal is modulated by a suitable sinusoidal or exponential function using the equation:

D = c/2f × φ/2π(1)

where: f—modulated frequency of reference laser signal; φ—phase of signal; c—velocity of signal.

For a precise measurement of distances, the laser beam is divided into three components with different modulation lengths. Thanks to the horizontal movement of the scanner head and the vertical rotation of the mirrors, it is possible, by considering the rotation angles counted by the scanner’s stepper motors, to calculate the *x*, *y*, *z* coordinates of the measured points, for which the value of the reflected laser light is additionally stored:

*x* = *d* × cosβ × cosα
*y* = *d* × cosβ × sinα
*z* = *d* × sinβ (2)

where: β—elevation angle of laser beam; α—laser beam direction angle in horizontal plane; *d*—distance from scanner to target point.

The set of all these points makes up the so-called “cloud” of points which represent the surfaces from which the laser beam was reflected. Each point in the cloud can also have an RGB colour value assigned to it, which corresponds to the colour recorded by the photos taken by the scanning device. The same points measured from different scanner locations serve to orientate the data obtained from several measuring stations, which are the so-called adaptation points for the spatial transformation. Most often, markers are reference spheres or discs but can be other uniquely identifiable elements of existing buildings (points, edges, and surfaces). The location of the scanner stations during the measurement session has an impact on the effectiveness of the measurement. This becomes especially important when using this method in the interiors of historic buildings, which are often cramped and have complex geometry. This makes it impossible to freely arrange the stations and signals as there is a risk of damaging the object. Measurement data were developed in several stages, first by registering scans from individual positions. This is to give the so-called georeference, using a transformation matrix, in the form of a uniform spatial coordinate system with the lowest possible statistical uncertainty. It is an iterative process that uses reference points (targets) and was performed with Z + F LaserControl 8.8.1 software. The designated cloud of points is used to perform analysis directly based on the designated spatial coordinates of each point in the cloud, and to create a solid model of the object. The point cloud analysis was based primarily on Cloud Compare and Surfer^®^ software, and the modelling was performed using Autodesk Revit^®^.

#### 2.2.2. The Use of TLS Technology in the Church

**Figure 5:**
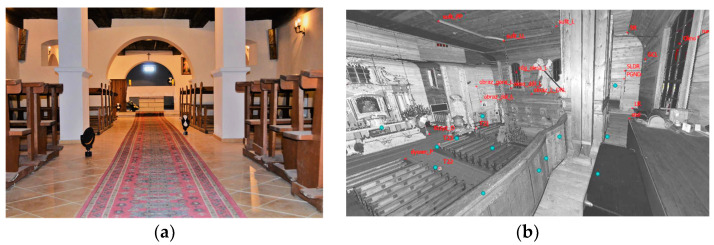
Measurement targets in the underground chapel (**a**) and the view of the scanning data in the reflectance intensity of the laser beam—one of the scanner positions inside the church (**b**).

**Figure 6:**
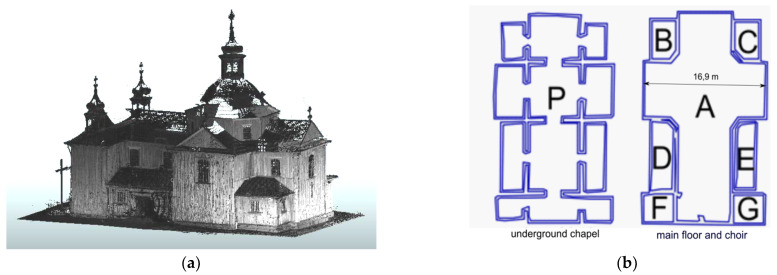
(**a**) Point cloud representing the church from the outside and (**b**) floor plan based on horizontal cross-sections through the point cloud.

It was planned to determine the geometrical relationships between the structural elements of the analysed construction. Since it is not possible to refer directly to all structural elements, it was assumed that the covered load-bearing elements would be represented by the analysed cover fragments directly attached to the structural elements. Because the wooden structure is erected on a brick basement, it was decided that the geometrical relationships of both types of structures should be defined in the form of the horizontality of the floors and the ceiling, the verticality of the bearing columns, and the verticality of the walls. Finding the relationship between these will allow the analysis of a potential common image of deformation, based on finding a similar trend. Additionally, analysis of the shape of the roof structure will strengthen any inference made. The created solid model (Revit application) is an additional element that will allow not only visualization of the church model but also preparation for a subsequent finite method analysis (FEM). The measurements carried out during the project were divided into three stages. At all stages, measurements were supplemented with digital photos that were taken using a built-in camera so that it would be possible to apply texture to the resulting point cloud later on. First, measurements were carried out in the underground brick part of the building (Figure 5). This level consists of 12 rooms. The second stage was the measurement of the main, wooden structure of the church and rooms on its level (Figure 3, Figure 4 and Figure 6) and two positions in the choir, as there are two additional rooms. The final stage was the measurement of the church from the outside.

A total of 30 point clouds were obtained from the measurements. The scans were combined into two stages. The first contained 22 scans made inside the building, while the second contained 8 scans made when measuring the exterior of the church. Satisfactory accuracy of a homogenous point cloud using several dozen scanner positions was obtained, characterized by errors in the position of the scanned points in the global system below ±10 mm. After performing procedures for filtering point clouds to take account of reflection quality and removing unnecessary elements and overrides, a uniform file was obtained containing scans made both inside and outside the building (Figure 6a), from which the outline of the rooms of the lower and main floors of the church could be separated (Figure 6b). The measurement of the external walls of the structure was supplemented with LIDAR data in order to supplement the point cloud with a part of the roof that was not available from ground measurements.

#### 2.2.3. Interferometric Method

Radar interferometry technology was used to verify the ground deformation data obtained from the measurements. InSAR (interferometric synthetic aperture radar) is a remote sensing method that uses the mutual phase shifts of the signal of two radar images taken on different dates. Based on the phase differences of the corresponding satellite radar signals, information about the relative values of the elevation of the terrain surface or its changes over time is obtained from the successive InSAR views. It is used to study changes in the shape of the Earth’s surface and to assess the location of structures. Buildings are most often classified as the so-called permanent scatterers, allowing for a good reflection of the emitted radar signal. When used for displacement analysis, we usually refer to the PSInSAR (persistent scatterers InSAR) method, which was used in this study. Similar analyses were performed by other authors as presented, for example, in [44,47,48].

#### 2.2.4. Finite Element Method Analysis

FEM is a method of numerically solving boundary problems [2], which is based on the applied multidimensional interpolation of the functions sought on a specific, discrete set of its nodes, the so-called finite elements. The quantities to be determined in FEM are the unknown ordinates of the interpolated function and its derivatives, occurring only in the subdivision nodes.

The FEM numerical analysis is effective if the structure is precipitated from the equilibrium position to the equilibrium position of the deformed structure. To this end, in order to bring the structure out of its nominal position, it is either loaded with small imaginary horizontal forces or directly forced to change the geometry of the system by slight deviations from this nominal position. The role of these displacements of the structure (determined by the geodetic measurements described in the article) is presented in the known equation of dynamic equilibrium:

(3) $$M\cdot \ddot{q}+C\cdot \dot{q}+K\cdot q=P\left(t\right)$$

where: *M*—mass matrix, *C*—damping matrix, *K*—elastic matrix, *P*—matrix of external loads as a function of time *t*, and *q*—displacement.

The main advantage of FEM is the ability to obtain solutions for areas with complex shapes, for which it is not possible to perform precise analytical calculations. FEM is widely used in the mechanics of construction and of continuous mechanics. With its use, the strength of the structure is tested, its deformations, stresses and displacements are simulated, thanks to which it is also possible to apply this method in the described problem [49,50]. Due to the complicated calculation process, special software packages are used for this. Autodesk Robot software was used in this study. After obtaining a solution, the results should be verified. In the case of a modeling error, we speak of a model validation. Such a validation is, for example, comparing the modeled displacements and deformations with those obtained in the course of geodetic measurements. Thanks to such validation, an answer was obtained about the main causes of deformation of the discussed structure.

## 3. Results

This chapter presents general data describing the building based on TLS measurements, specific deformations of structural elements, and analyses based on PSInSAR satellite studies and FEM modelling. All studies allowed to determine the causes of deformation of the object using only non-invasive measurements and analytical processes.

### 3.1. Church Measurements

**Figure 7:**
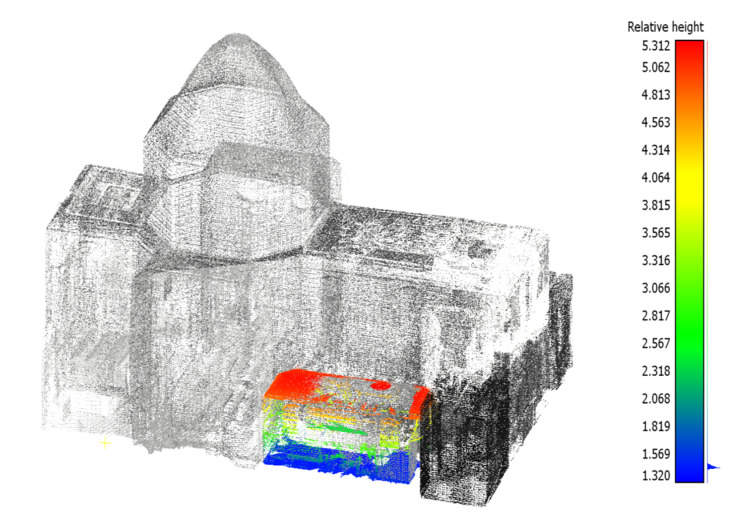
Points used to measure the built-up area, volume, and height of room D (on the right—hypsometric legend of relative height of objects (m)).

According to the analysis, the church is 27.75 m high, has a total area of 695.50 m^2^, and is 4150 m^3^ in volume. Room volumes were determined based on the area determined in AutoCad 2020 and the average height based on the floor and floor identification using CloudCompare. An example of the analysis conducted for room D is shown in Figure 7, and detailed results for individual rooms are presented in Table 1.

**Figure 8:**
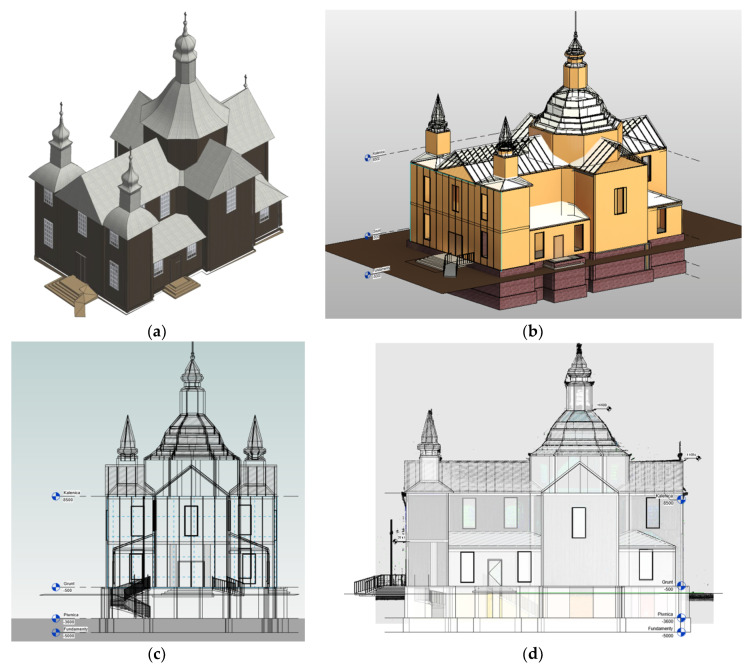
Model of the church St. Szczepan in Mnichów—southwest view ((**a**)—rendered model and (**b**)—structural model) and construction projections ((**c**)—view from the entrance and (**d**)—view from the south-west).

Based on the work carried out, a virtual replica of the building was created in the form of a 3D model, which is shown in Figure 8. In order to reproduce the colour of the outer walls of the church, RGB colour obtained from simultaneously taken photos was used.

### 3.2. Analysis of the Geometric State

**Figure 9:**
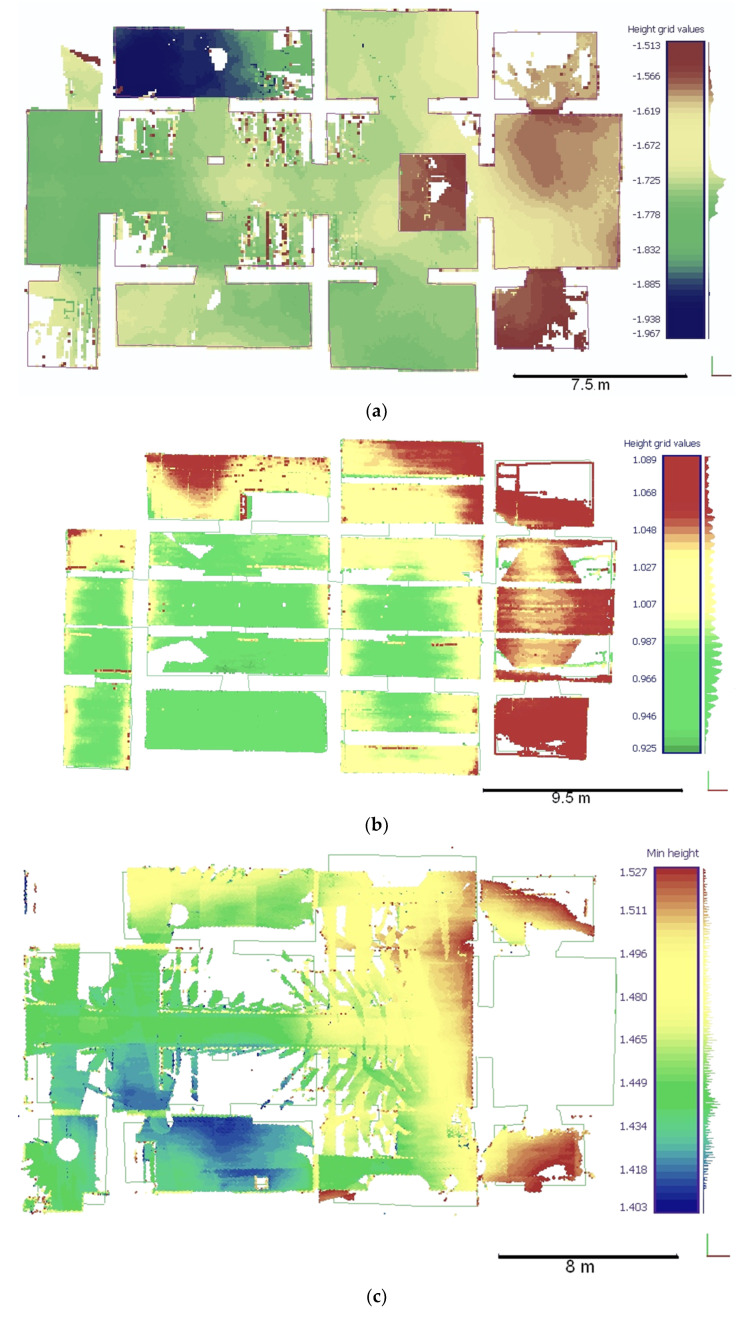
Relative changes (m) in floor height (**a**)—the underground chapel, (**b**)—its ceiling, and (**c**)—the floor of the nave.

In order to verify the assumptions about the compliance of the structure geometry with the theoretical model, a number of analyses were performed based on the acquired uniform point cloud. Among other things, an analysis of the floor level of both floors was carried out, based on the measured points after the filtration process (removal of building elements) and thinning of the cloud. A matrix of points of the tested surfaces at regular intervals of 0.05 m was obtained, which was then subjected to the process of determining the floor height changes in the CloudCompare program and controlling the interpolation of the contour lines in the Surfer^®^ program using the Nearest Neighbours algorithm. Figure 9 presents hypsometric plans for the level of the floors in the rooms of the underground chapel (Figure 9a) and its ceiling (Figure 9b) using the system adopted for local height.

The map presented in Figure 9a shows the differences in the level of the individual floor surfaces in the underground chapel. Unfortunately, due to the status of the monument, it was impossible to make measurements of covered structural elements (foundations), but these level variations, which are within the limits of 5 cm in individual rooms, can be considered statistically significant. The same conclusion can be drawn from the examination of the level of the underground chapel ceiling (Figure 9b). Similar procedures were also carried out for the nave floor (Figure 9c). Due to the presence of large items of content that could not be removed, and the fact that the boards covering the floor are only a masking rather than a structural element, the height difference determined may not reflect the level of the floor with complete accuracy. However, it does show a south-west slope, which is also indicated by the ceiling of the underground part.

**Figure 10:**
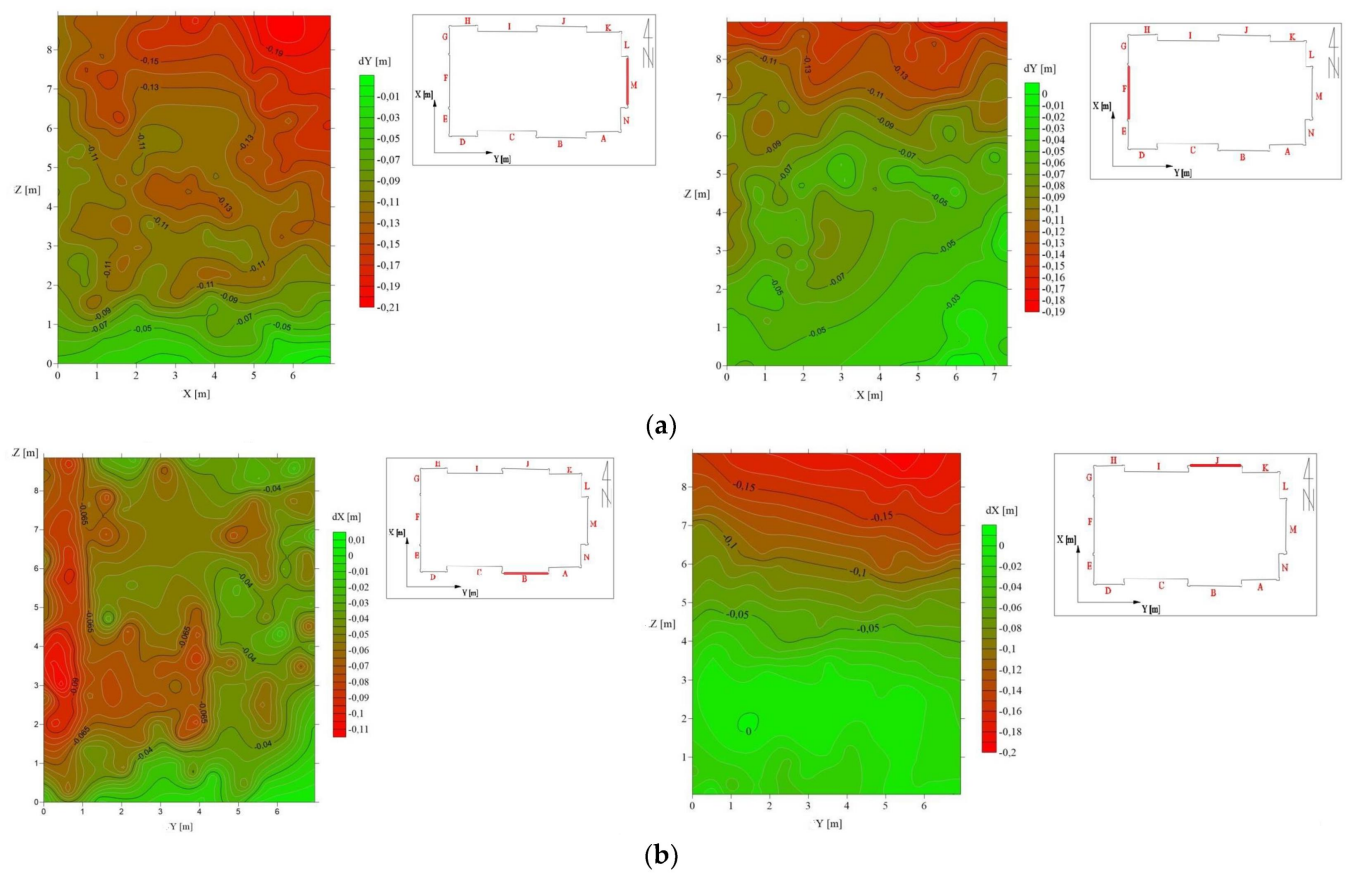
Deviations from the vertical planes of the external walls of the structure—(**a**) walls ”M“ and “F” on the west-east axis and (**b**) walls ”B“ and “J” on the north-south axis.

An important aspect of the stability of a wooden structure is the verticality of its walls. Deviation from this condition may place areas at increased risk of stress transmission and the worsening of conditions for structural statics. The church has 14 external wooden walls, and these were subjected to verticality analysis. The designations of the walls are shown in Figure 10. As in the floor level test, points belonging to the individual walls were selected and points corresponding to window and door openings were removed. Then, the point cloud was thinned out so that the minimum distance between points was 0.5 m. In the evaluation of the contour plans, local systems were used, consistent with the spatial orientation of the examined plots. The axis perpendicular to the axes delineating the wall planes was taken as the coordinate indicating non-verticality. It was found that the walls of the building are tilted west (towards the main entrance, opposite the altar) within (6 ÷ 34 mm/m) and to the south within (9 ÷ 21 mm/m). Sample charts of vertical line isolation are presented in Figure 10 and the data is detailed in Table 2.

The determined values of deviations from the vertical mean at a height of about 9 m; the deviation of the walls from the vertical can reach as much as 30 cm. One resultant value cannot be directly indicated here because these walls are not connected in one monolith. Nevertheless, all the vertical deviation values indicate a south-west slope of the structure. Figure 10 shows the deformation of the walls with zones that correspond to the relationships between the nodes of the frame structure. Thus, the identification of these zones indirectly indicates the type and degree of deformation of the church structure. It should be remembered that the church was built in the 18th century from larch wood, which is considered to be among the most durable and resistant to deformation caused by weather conditions and the passage of time. The slope of the walls could, however, be influenced by the way the building was erected and the lack of precise technology for preparing wooden structure elements.

**Figure 11:**
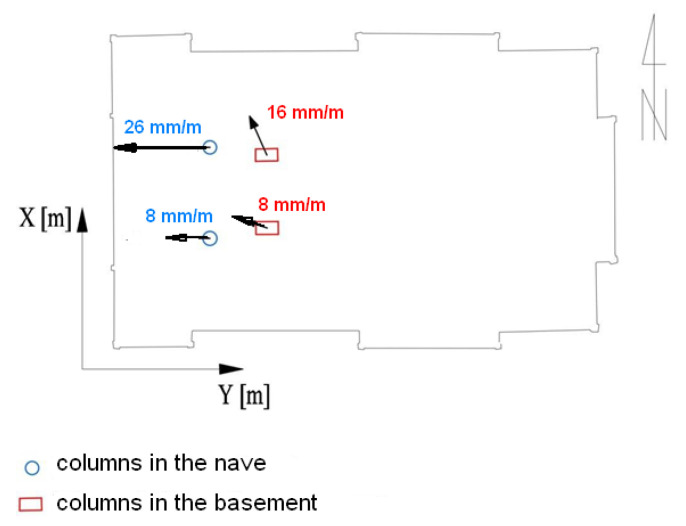
Determined vectors of inclination of the column axes from the vertical.

One of the elements of the internal structure which exhibits potential deformation is the columns supporting the floor on both floors. In the main nave they support the choir (Figure 4), while in the underground chapel they support the ceiling (Figure 5). To determine the values of deflection from the vertical of the columns, it was necessary to determine their height, as well as the values of the shift of their centres in the horizontal plane. The assessment was based on some extreme sections, for which the geometric centres of each column contour at individual height levels were determined. The cross-sections of the columns were separated from the obtained point cloud, which enabled analyses based on the Cartesian coordinates of these points. The results of the calculations are presented below in the Figure 11.

**Figure 12:**
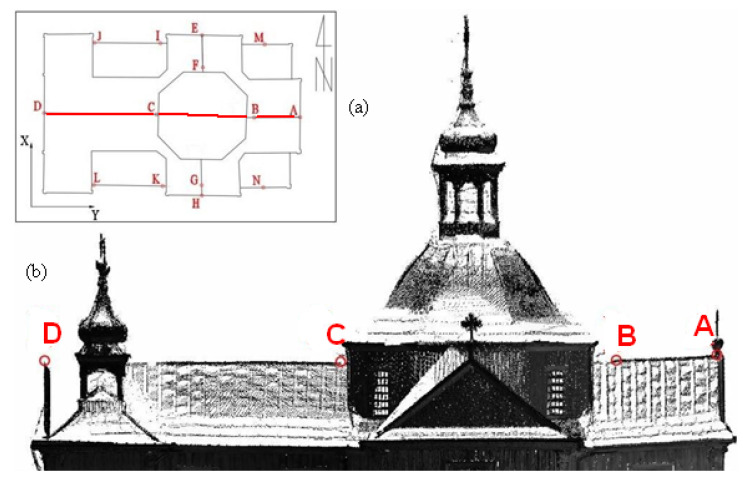
View of the ridge of the church roof, west-east ((**a**)—view from the top and (**b**)—side view).

The columns in the underground chapel are again tilted to the north-west. The northern column has a greater inclination, i.e., 16 mm/m. The columns located in the nave of the church lean only to the west. The northern column has a greater inclination, i.e., 26 mm/m. These analyses also indicate that the inclination of the structure at the level of the main nave is predominantly in the western direction, similar to the external walls of the structure. The structure of the church is reinforced by a roof truss, which interacts with elements of the wooden skeleton in a much more active way than in modern concrete and brick constructions. For this reason, checking the shape of the truss, by examination its symmetry, may allow additional inferences to be made regarding the condition of the entire structure. For this purpose, analysis of the levelness of the roof edges (ridge) in the longitudinal and transverse directions and of the roof slope inclination were performed. The ridge slope was calculated based on the distance between the characteristic points (Figure 12) and the height difference between them using trigonometric functions.

**Figure 13:**
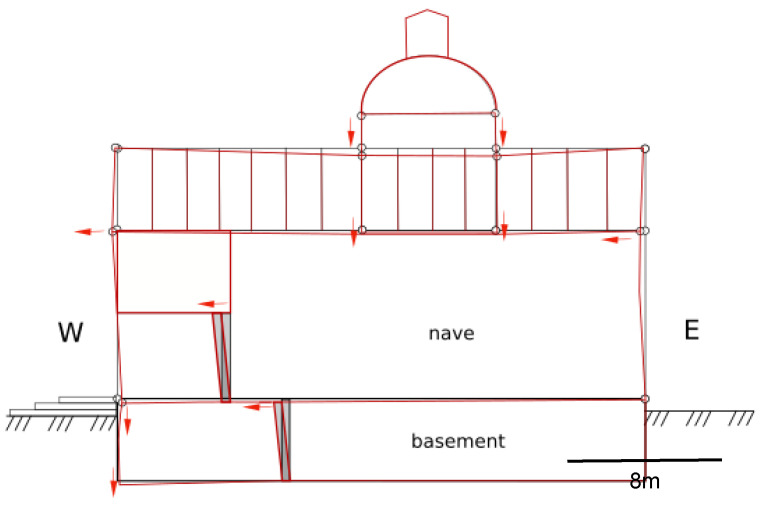
View of identified displacements of church structure.

Maximum gradients were found in the B–A section (+38 mm/m) but also in a north-south direction for the E–F section (−40 mm/m). Roof slopes analysed in cross-section indicate warping and torsion of their planes but not exceeding ±1.4% (up to 70 mm/5 m). This indicates that the roof structure has also undergone deformations caused by the subsidence in the foundation zone described above. A general image of the determined displacements of the church structure is shown in Figure 13. It shows structural deformations as a logical whole, probably caused by the instability of the soil under the south-west part of the foundations, which translates into the reaction of the entire wooden structure at its nodes.

**Figure 14:**
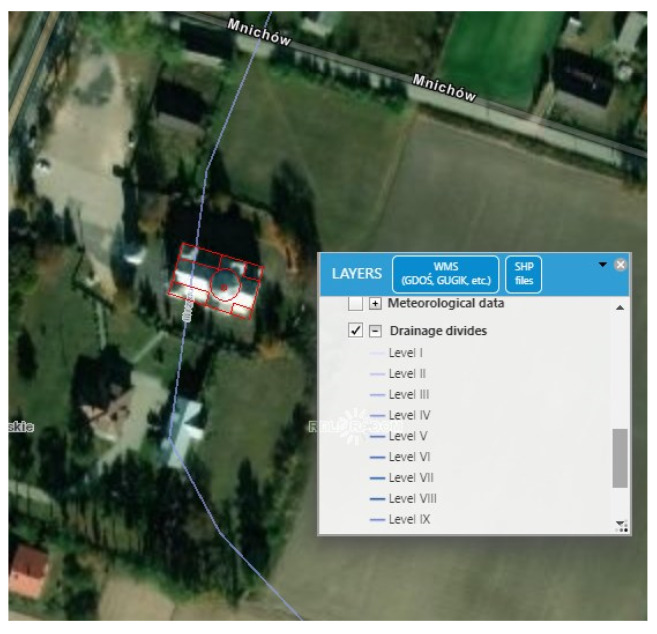
Course of border of third level watershed (drainage) near church (source: https://www.bdl.lasy.gov.pl/portal/mapy-en, accesed on: 20 October 2021).

Hydrogeological information concerning the site’s foundation area was also analysed. It was found that a water division line runs through the western part of the structure (Figure 14). The information presented on this map confirms that there may be an influence of various water conditions in the ground beneath the church, which translates into the observed consistent image of the deformation of the structure, which cannot be explained by construction imperfections and/or natural degradation of the structure. However, even such large deformations as those described here have not caused changes in the form of scratches, cracks or delamination, which could be noticed visually and would signal the need for quick preventive measures. (Note that such changes would be almost immediately visible in brick or stone as opposed to wooden structures.) As the results above indicate, this lack of symptoms of deformation does not mean that the structure of the church is in good condition but is rather indicative of the quality of the material that was used in construction. This means that due to the very high probability of increasing of the deformation, further control and diagnostic tests had to be performed to confirm the cause of the deformation non-invasively.

In order to verify the thesis about the influence of variable water relations in the ground on the deformation of the church structure, a study was carried out with the use of radar interferometry in the PSInSAR variant [51], the aim of which was to show heterogeneous subsidence of the terrain and structure over a longer period (1 year). Modeling was also performed with the use of the finite element method, in which the values of displacements found from geodetic measurements and the InSAR method were used to calibrate the resulting model and to determine the deformation model as close as possible to the actual ones.

### 3.3. PSInSAR Analysis

The study area is limited from 50°38′ N to 50°45′ and 20°17′ to 20°27′. In this study, the Sentinel-1A data in the Single Look Complex (SLC) format are utilized. The dataset is acquired in the descending orbit number 51 and is processed in the interferometric wide-swath (IW) mode. We used a total of 30 images covering the 1-year time period, between 4 January 2019 and 30 December 2019, with a 12-day temporal interval. The radar wavelength of Sentinel-1 is ~5.55 cm, which corresponds to the C-band in the microwave spectrum. The image captured on 28 May 2019 was chosen as the primary image.

**Figure 15:**
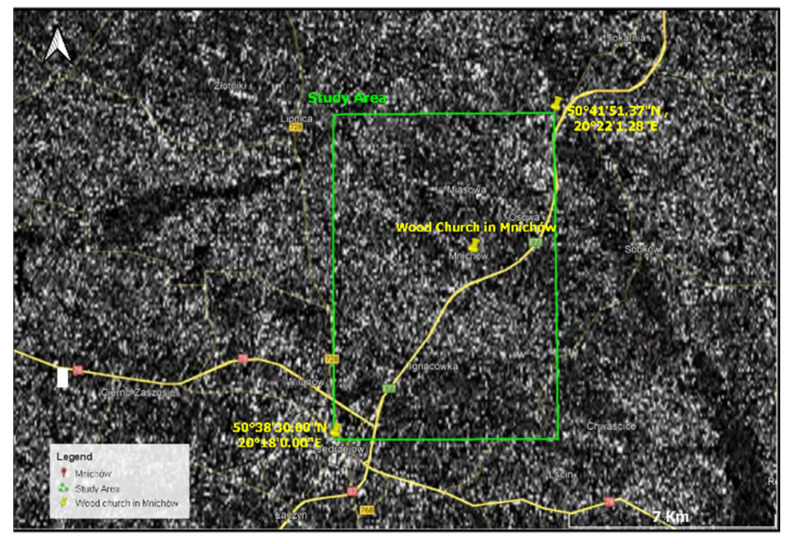
The raw interferogram picture of coherence (value of coherence from 0 to 1). From 0.4 to 1 is good coherence (white colour) and near to 0 is bad (no satellite signal or bad value of signal) and has black colour. The standard filtered coherence value > 0.4 was used in the calculations.

The Sentinel-1A data is processed with the PSInSAR method. The interferograms are first processed by the Sentinel Application Platform (SNAP) software package by European Space Agency (ESA), then the InSAR time series are analysed by the Stanford Method for Persistent Scatterers (StaMPS) software package (Figure 15).

**Figure 16:**
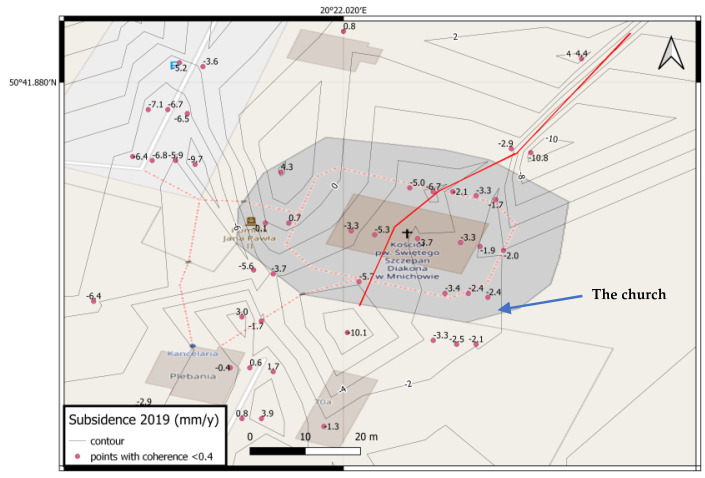
Annual ground depressions (grey contour lines and red points) near the church observed in the period January—December 2019, corresponding to the date of geodetic measurements. Red line—observed division on two regions crossing the church building.

As a result of the development of the PSInSAR technique, the result of annual vertical displacements of the terrain in the area of the analysed church building was obtained, which was visualized in the form of contour lines (isocatabases) generated on the basis of clearly identifiable points of high coherence. The results are shown in Figure 16. The red line also shows the division into two regions, the boundary of which intersects the building, in accordance with the previously presented hypothesis about the watershed (Figure 14).

### 3.4. FEM Analysis

In order to qualitatively verify the observed phenomenon of deformation of the church structure, modeling with the finite element method was used. Several 2D models were created in Autodesk Robot that represented a cross-section of a structure with elements representing individual parts of the structure. The modeling version closest to the actual shape of the structure found by geodetic measurements is presented below, in which the foundation is modeled as a bar, on it the basement walls as columns, the bottom of the nave as a bar, the nave structure as columns, and the ridge as a girder. The foundation and basement were defined as concrete (closest to the probable form as bonded stone) and the remainder of the structure was actually defined as timber. The supports are defined on the foundation. The model also takes into account the results obtained from the remote sensing analysis by the radar interferometry (PSInSAR) method, which clearly indicates the existence of dividing lines into zones with different depressions in the ground (Figure 16). It coincides with the line of the watershed found on hydrogeological maps, which makes the thesis about the primary cause of the structure deformation overlapping with successive secondary factors more probable.

**Figure 17:**
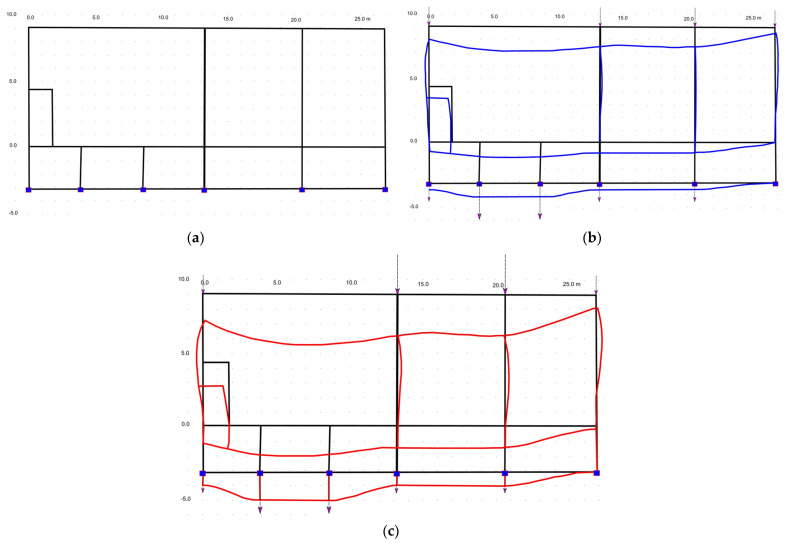
Deformations of the structure of the basement ceiling and the nave, confirmed by modeling, taking into account the determined condition from geodetic measurements and PSInSAR analysis: (**a**)—theoretical model of the basement and the nave; (**b**)—structure deformation (blue lines) under the influence of changing water relations in the soil; (**c**)—deformation of the structure (red lines) due to the total changes in water conditions and additional load (arrows) on the structure of the church tower in the center of the building.

The preliminary models, which did not take into account changes in the soil, which were based only on the strength conditions of the wooden structure erected on a stone foundation, indicated only the load of the central part of the nave and the lowering of the roof in the middle part. This explained the overload of the structure in this area, but did not explain the reasons for the inclination of the floors in the underground part of the church and the deviation of the column supports in both storeys in the same direction, in line with the slope of the ceiling and floor of the lower chapel. Only taking into account the information about the instability of the soil under the influence of water changes, which was also detected as well as confirmed by InSAR tests, made it possible to adjust the FEM modeling to the state determined by in situ tests. The next model assumed the influence of the heterogeneity of the foundation setting but without overloading the roof structure (Figure 17b). This model also did not present the complete picture of deformation, although it was already closer to reality.

As a result of multiple FEM analyses, the following (Figure 17c) deformation shape of the structure elements in the underground and above-ground parts of the church was obtained. The best-fit model may be obtained for uniform ground subsidence on the west side of the building; however, any increased subsidence in a center of the church will pull this asymmetric structure to the east. It follows that the condition of the structure determined by measurements may undergo constant changes and the deflection of walls and supports may change, depending on the intensity of revealing depressions in the ground resulting from changes in water relations in the area of the facility. The additional load in the floor part increases the deformation phenomenon, which explains the greater deformations found that would result from the influence of variable water relations in the soil. Modeling also showed a significant influence of overloading the structure supporting the tower, which additionally influenced the settlement of the central part of the building. The combination of both of the above-mentioned factors (changes in water relations and overload) led to the found image of deformation, detected by non-contact geodetic surveys. Thus, the assumed thesis on the influence of factors deforming the structure on the basis of non-destructive tests was confirmed. The detected process is continuous and variable over time depending on the current water conditions in the ground. At the same time, the second factor identified by FEM modeling will continue to adversely affect the behavior of the structure. Both reasons indicate the need to continue the research on a regular basis as part of the monitoring of the historic church.

## 4. Discussion

The scope of the analysis of the geometric condition of the historic church above represents a significant portion of possible research. However, under conditions where a historic building is used daily for religious activity, it is difficult to interfere directly with or disassemble a structure or its contents even on a temporary basis for the purposes of further research. For this reason, using measurements based on laser scanning seems to be the optimal method, guaranteeing an appropriate level of accuracy of the survey performed. This measurement technique is based on reference points (targets) that allowed the proper and unambiguous creation of a 3D model of the entire church. Note that “targets” could not be used in all conditions. It was necessary to use algorithms that made it possible to combine the measurement stations into one model using the “cloud to cloud” procedure. In this way, there was no need to use reference points and the merging was based on automatically determined dependencies between points, planes, and edges detected by the algorithm [52,53] of the analysed scans. In the study above, it was possible to confirm that by using the appropriate measurement regime (based on some fragments of the scanning of the church building), a model can be made at a similar level of accuracy as with the use of targets. Due to the possibility of registering additional attributes in assessing the structure, apart from an RGB camera, an infrared camera can be used, which will allow the assessment of the energy stability of the facility. This is very important from the point of view of preventing the degradation of the structure and contents of such a historic building, as it allows the detection of the so-called thermal bridges showing areas of heat transfer through the structure. In this study, the author did not have access to a device such as this, but the legitimacy of using such a research method in the evaluation of historic buildings is clear.

A wooden church is a particularly difficult candidate for the analysis of geometric state. This is because wood is susceptible to the transmission of deflections and deformations even under conditions of large deformation. Therefore, it is difficult to determine safety thresholds and each structure should be examined on a case-by-case basis. The present results of the assessment of geometric condition by means of laser scanning allow us to draw conclusions about the relationship between the parallelism, flatness, horizontality, and verticality of the structural elements, which in most cases gives a sufficient basis for making inferences about the safety of the building. It was found that the columns deflect from the vertical by 8 to 26 mm/m towards the west, the wooden external walls are deflected 6 to 34 mm/m also towards the west, and the floors of two levels have a subsidence of 5 cm on the west side. The roof also shows deformations, as indicated by ridge inclinations of up to 40 mm/m. However, as mentioned earlier, such deformations are not immediately visible in wooden structures, as demonstrated in this article. Therefore, in such cases, it cannot be unequivocally stated, as it is in articles on concrete engineering and brick structures [38,54], that an increased load on the beams of the structure appears almost immediately in the form of deformation and is additionally included in the functional relationship.

The article demonstrates, by means of a case study on the form of a wooden monument, that the non-contact diagnostic method is sufficient to obtain a complete picture of structural deformation. The described method of diagnostic development additionally enables other studies to be carried out on the existing digital model. Due to the possibility of combining the effects of deformation into a uniform, simplified picture of changes, the diagnostics of structure geometry can become the basis for FEM analysis.

The church has not been rebuilt since its erection, and despite the imperfections identified in the article, at the beginning of 2014, only a new covering was made. However, this study suggests that further interventions may be needed in the future, if and when the observed deformations continue to increase.

## 5. Conclusions

St. Szczepan’s Church in Mnichów is about 250 years old. This means that it is in fact a relatively young monument. However, due to the material from which it was built, and the technology used, the structure is less durable than other architectural buildings made of brick or stone. For this reason, the fact that it has stood for more than two centuries without renovation is impressive. The structure of the church is notable for the high accuracy of its execution, especially bearing in mind the standards of equipment available at the time of construction. To carry out the survey, laser scanning was used as a measurement technology. This made it possible to conduct non-invasive tests of the geometrical state of the entire structure without interfering with the arrangement of the structure’s elements. The laser measurements, together with the simultaneously conducted photographic session, allowed the creation of both geodetic and architectural documentation and digital preservation of the current state of the monument. Certainly, digital spatial models in the form of a point cloud and a solid model are also helpful in this respect, allowing for multiple tests and comparison of their results in the case of periodic surveys.

The methodology proposed in this article, carried out in conditions of limited accessibility to the building, allowed for the correct determination of the deformation state of the church structure and inferences about its causes. Current geometrical condition showed some existing imperfections in the shape of the church structure, the surface of the floors, walls, and roof slope. These are due partly to imperfections in the construction methods of more than 200 years ago, but also to the influence of external conditions, probably related to inhomogeneous water relations in the soil. This hypothesis is confirmed by the dominant direction of the deviation of the structure towards the west, as determined in several analyses (FEM, PSInSAR).

Regardless of the deformations identified in the building’s geometry, the condition of the church should be considered relatively good, even though in recent years the building has not undergone any renovation works that might retard the changes in its condition and geometry. Due to the historical value of the monument, periodic re-measurements are planned in order to check whether the church’s geometry will change further. This is all the more important because on the basis of the studies performed, it cannot be clearly stated when the changes in the geometry of the church took place over time, as there is no documentation of measurements from previous years. Despite the strong verification of the hypothesis about the instability of the subsoil on the south-west side of the facility, GPR surveys are planned to assess the structure of the subsurface layers of the ground, which will confirm the need for drainage around the church and the construction of piezometers to monitor the groundwater level.

## Figures and Tables

**Table 1:** Geometric characteristics of the rooms of the church.

Room	Circumference [m]	Height [m]	Area [m^2^]	Volume [m^3^]
P (underground)	221.3	3.47	320.87	1113.42
A (main nave)	83.91	17.77	260.17	2556.3
B	16.66	4.18	17.5	73.16
C	16.1	3.75	16.34	61.26
D	23.17	3.99	26.7	106.53
E	21.65	3.94	21.64	85.25
F	16.13	4.76	16.19	77.06
G	16.04	4.62	16.01	73.97

**Table 2:** The value of the wall deviation from the vertical (wall markings in accordance with Figure 10).

Wall	Number of Points	Height of Walls (m)	Slope Value (mm/m)	Tilt Direction
A	49	3.63	21	South
B	160	8.85	19	South
C	63	3.67	13	South
D	94	8.78	9	South
E	45	8.73	6	West
F	150	8.97	19	West
G	74	8.68	17	West
H	75	8.93	9	South
I	60	3.30	17	South
J	151	8.76	16	South
K	46	3.51	11	South
L	24	3.43	26	West
M	162	8.87	35	West
N	95	3.52	34	West

## Data Availability

The datasets used and/or analysed during the current study are available from the author on reasonable request.
